# Cost, context, and confounding: rethinking real-world data generation and interpretation in advanced NSCLC

**DOI:** 10.1093/oncolo/oyag106

**Published:** 2026-05-04

**Authors:** Mehmet Mutlu Çatlı, Arif Hakan Önder

**Affiliations:** Department of Medical Oncology, Antalya Education and Research Hospital, Antalya 07090, Turkey; Department of Medical Oncology, Antalya Education and Research Hospital, Antalya 07090, Turkey

**Keywords:** real-world data, immunotherapy, cost-effectiveness, NSCLC, rechallenge

## Abstract

**Background:**

As immunotherapy becomes entrenched in the frontline management of advanced NSCLC, post-progression treatment decisions face increasing uncertainty. A recent JAMA Network Open study using Flatiron Health data suggested that rechallenging with pembrolizumab may offer survival benefit after chemo immunotherapy progression. However, these findings raise fundamental questions about how real-world data (RWD) should be interpreted and operationalized in health economics.

**Main argument:**

In this viewpoint, we critically analyze the methodological and interpretive assumptions behind retrospective RWD-based cost-effectiveness modeling. We argue that unadjusted survival metrics absent stratification for PD-L1 status, tumor burden, or clinical performance may artificially elevate ICER/QALY estimates and misguide reimbursement decisions. Using the Velcheti et al. study as a case anchor, we highlight how structural biases in RWD can lead to premature or inflated conclusions about the value of immunotherapy rechallenge. We also propose a visual framework to distinguish between plausible clinical signals and policy-grade evidence.

**Implications:**

Health systems and stakeholders must apply greater rigor when translating RWD into economic models. We advocate for analytic transparency, pre-registered sensitivity analyses, and adjustment for patient-level variables before using RWD to inform value-based oncology.

**Conclusion:**

RWD are powerful hypothesis generators, not substitutes for controlled evidence. Without methodological discipline, their policy application risks becoming speculative rather than strategic.

Implications for Practice The increasing use of real-world data in oncology has important implications for clinical decision-making, health technology assessment, and reimbursement policy. Clinicians and policymakers should be cautious when interpreting survival benefits derived from retrospective observational datasets, particularly when key prognostic variables such as PD-L1 expression, performance status, and tumor burden are incompletely captured or adjusted. Economic models based on such data may provide an appearance of precision while masking underlying clinical uncertainty, potentially leading to suboptimal resource allocation. Strengthening analytic transparency, preregistering sensitivity analyses, and improving the clinical granularity of RWD sources are essential steps to ensure that real-world evidence informs, rather than misguides, value-based cancer care.

## Introduction

Real-world data (RWD) has emerged as both a promising tool and a potential risk factor in contemporary oncology. Its appeal lies in the ability to capture broad, heterogeneous patient populations and to provide rapidly accessible insights into treatment outcomes outside the constraints of randomized clinical trials. However, when RWD is used to infer therapeutic value or cost-effectiveness without adequate methodological rigor, it may lead to what can be termed a probabilistic trap: the illusion that observed survival differences reflect treatment efficacy, while in fact they may be driven by unmeasured confounding or invisible selection bias.^1^^,^^2^

This dilemma becomes particularly salient in the setting of advanced non–small cell lung cancer (NSCLC), where clinicians increasingly face decisions about immune checkpoint inhibitor rechallenge following progression on first-line chemoimmunotherapy. A recent retrospective analysis by Velcheti et al., using the Flatiron Health database and published in JAMA Network Open, evaluated 1747 patients with advanced NSCLC and reported that post-progression pembrolizumab based regimens were associated with longer survival compared to either chemotherapy alone or immunotherapy monotherapy.^3^ Although Velcheti et al. included PD-L1 in subgroup analyses, the primary comparative analysis of pembrolizumab rechallenge versus chemotherapy was not stratified by PD-L1 expression, ECOG performance status, or tumor burden, which limits the interpretability of the observed survival differences. These variables are well known to influence both treatment selection and survival outcomes.^4^

Moreover, this is not an isolated instance. Across oncology, RWD studies that bypass key clinical and biological stratifications often proceed to inform incremental cost-effectiveness ratio (ICER) or quality-adjusted life year (QALY) calculations. When such unadjusted survival estimates serve as the basis for cost-effectiveness modeling, there is a risk of overstating value or misinforming reimbursement policies.

The goal of this viewpoint is not to dismiss RWD. Rather, we argue for a more nuanced and methodologically grounded interpretation, particularly in contexts such as immunotherapy rechallenge where clinical equipoise, selective patient eligibility, and value-based care converge. We will examine the methodological fault lines underlying real World immunotherapy rechallenge data, identify structural blind spots, and propose interpretive safeguards that should be met before such data can responsibly inform clinical or economic decision-making.

## Methodological fault lines in immunotherapy rechallenge RWD analyses

The appeal of real-world evidence lies in its scale and immediacy, but this very breadth can obscure meaningful heterogeneity if not rigorously stratified. In the case of immunotherapy rechallenge following progression on chemoimmunotherapy, retrospective data analyses such as that by Velcheti et al. may suffer from unmeasured prognostic imbalance. The reported survival advantage with pembrolizumab-based regimens could reflect not a treatment effect but rather pre-treatment selection, including better ECOG performance status, higher PD-L1 expression, or lower tumor burden in the immunotherapy group variables not adjusted for in the analysis.^3^ This raises the possibility of channeling bias, where patients with inherently better prognoses are more likely to be steered toward a particular therapy, creating a misleading association between treatment and outcome.^5^ Moreover, the absence of balancing methods such as inverse probability weighting or high-dimensional propensity scoring limits the credibility of comparative interpretations. As Booth et al. emphasized, real-world data in oncology may misrepresent treatment effects if baseline prognostic imbalances are not transparently accounted for.^6^

In addition to clinical stratification gaps, the structural properties of commercial RWD platforms pose analytical challenges. The Flatiron Health database, while widely used, does not systematically capture key clinical variables such as metastatic burden, brain metastases, or immunotherapy-related toxicities, and often lacks consistent documentation of treatment intent.^7^ Without understanding the rationale behind immunotherapy rechallenge versus chemotherapy selection, survival comparisons may confound clinical aggressiveness with therapeutic efficacy. Moreover, variable follow-up durations and incomplete event ascertainment introduce additional noise. Importantly, patients rechallenged with immunotherapy may represent a biologically selected subgroup, those who had longer responses or immune-sensitive tumors further biasing the observed outcomes. As articulated by Desai and Franklin, failure to incorporate these latent clinical variables can distort effect estimation in ways that undermine causal inference in RWD studies.^8^

These methodological vulnerabilities underscore the need for interpretive caution before translating real-world immunotherapy rechallenge outcomes into health economic value judgments.

## When economic clarity masks clinical ambiguity: the trap of misleading certainty

The integration of RWD into health economic models adds a layer of complexity to decision-making in oncology. On the surface, it offers pragmatic advantages mirroring treatment patterns in routine care and generating cost-effectiveness estimates that may inform reimbursement decisions. However, when underlying clinical variables are inadequately reported or stratified, metrics such as QALY or ICER can create an illusion of precision, producing models that obscure rather than clarify clinical value.

This concern is particularly visible in the recent retrospective analysis by Velcheti et al., which evaluated 1747 patients with advanced non-small cell lung cancer (NSCLC) using the Flatiron Health database.^3^ The study observed longer overall survival with pembrolizumab rechallenge compared to either chemotherapy alone or monotherapy checkpoint inhibition following chemo-immunotherapy progression. While the findings appear favorable, they are derived from a dataset lacking stratification by PD-L1 expression, tumor burden, performance status, and molecular profile critical variables that define clinical context. Transferring such survival results directly into QALY or ICER frameworks risks producing overstated or distorted estimates of treatment value.^6^^,^^9^^,^^10^

We refer to this phenomenon as the “trap of misleading certainty.” When survival gains are modeled without adequate adjustment, especially in populations with favorable baseline characteristics more likely to receive immunotherapy rechallenge, the economic outputs may misrepresent true comparative effectiveness. In this case, the high QALY or favorable ICER derived from RWD may be less a reflection of therapeutic merit and more a function of confounding.

**Figure 1.**
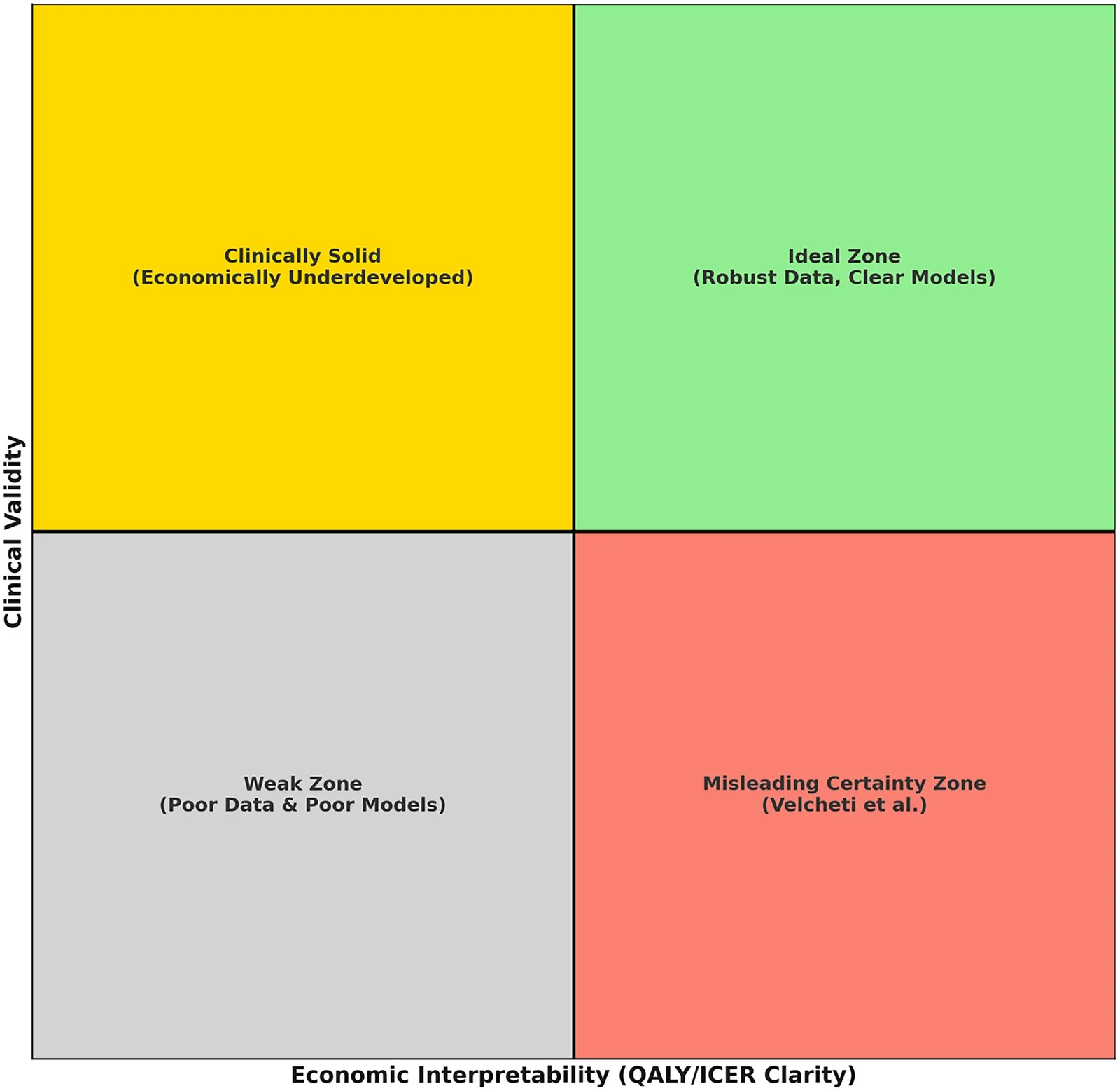
The certainty–validity framework for interpreting RWD-derived economic models. RWD, real-world data.

To illustrate this mismatch, we propose a quadrant-based conceptual framework (Figure 1). The Y-axis captures the clinical validity of the underlying data, while the X-axis reflects the economic interpretability through QALY/ICER modeling. The upper right quadrant represents ideal scenarios, robust data with sound economic extrapolation. The lower left quadrant, where we place the Velcheti analysis, reflects a problematic zone: models that appear economically strong but are built upon clinically underpowered or unadjusted evidence. This is the epicenter of misleading certainty.

The lower left quadrant captures a critical tension in contemporary oncology economic models that offer clarity without clinical integrity. The placement of Velcheti et al. within this quadrant reflects limitations in clinical granularity (eg, incomplete adjustment for performance status, tumor burden, and treatment selection factors) rather than a judgment of overall study quality.

The implications are far from theoretical. When such models are used to support reimbursement decisions, design value frameworks, or shape clinical guidelines, they risk distorting resource allocation and misguiding treatment strategies. Past experiences with second-line and third-line NSCLC therapies have demonstrated how modest observational survival differences amplified through economic models can influence access decisions disproportionately.^11^^,^^12^

These risks are amplified when the data sources and their commercial relationships remain opaque. Flatiron Health is a privately owned data platform, and GlaxoSmithKline (GSK), the study sponsor, is a pharmaceutical stakeholder in the advanced NSCLC space. This arrangement does not inherently invalidate the data but heightens the ethical obligation for methodological transparency, adjustment for confounding, and disclosure of analytic limitations.^13^

In summary, economic clarity must not be allowed to mask clinical ambiguity. While RWD can advance value-based care, its utility depends not only on what is modeled but also on how and why it is modeled. As these datasets increasingly shape policy and practice, oncology must remain vigilant to distinguish analytical strength from seductive but misleading precision.

## Value claims and the risks of overtranslation

Once real-world findings are extracted from retrospective datasets, the act of interpretation becomes one of accountability, not merely analysis. These findings have potential clinical relevance but raise important concerns when extended into the economic sphere.

Although the authors did not directly calculate QALY or ICER metrics, the structure of their analysis has clear implications for downstream cost-effectiveness modeling, particularly in contexts where unadjusted outcomes are used as raw inputs for QALY modeling in industry-sponsored economic analyses.^9^ When overall survival benefit appears superior in one group, even with incomplete adjustment for baseline risk factors like ECOG performance status, tumor burden, or PD-L1 expression, it risks skewing economic assessments in favor of that group, especially when data are used to infer treatment value that may influence public payer decisions.^14^^,^^15^

This is not a hypothetical risk. Real-world data are increasingly incorporated into health economic evaluations submitted to agencies such as NICE in the UK, HAS in France, and PBAC in Australia, particularly when randomized trial data are lacking or limited.^16^ However, these data must meet clear methodological standards and be accompanied by transparent documentation of confounding adjustment strategies. Without such rigor, economic models built on retrospective observational inputs may generate misleading cost-effectiveness conclusions, especially in high-stakes oncology reimbursement settings.

**Figure 2.**
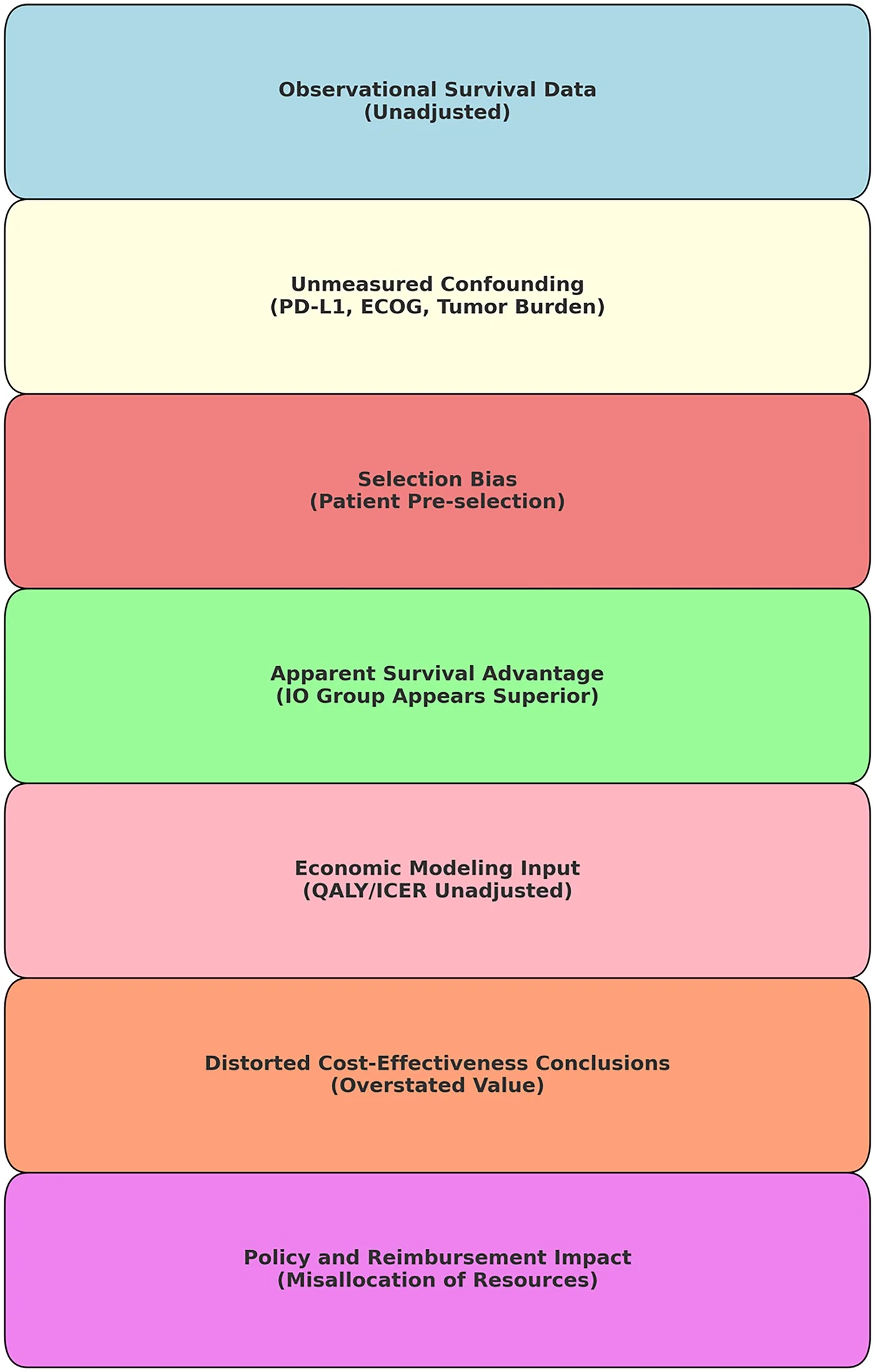
Bias propagation from RWD to economic models (vertical layout). RWD, real-world data.

The challenge is not merely technical. Even sophisticated adjustments cannot fully correct for latent confounding in retrospective oncology datasets, particularly when treatment selection is non-random and patient characteristics are incompletely captured.^17^ In this context, the selective survival advantage observed in the pembrolizumab rechallenge group may partly reflect favorable baseline prognostics rather than true therapeutic superiority. Figure 2 schematically illustrates this disconnect, showing how observational survival curves when translated too directly into economic modeling can propagate systematic bias across decision frameworks. Such errors are difficult to detect once embedded in formal QALY or ICER calculations and may ultimately misinform clinical guidelines or reimbursement pathways.

Hence, the threshold for accepting real-world survival comparisons as the basis for value claims must be higher not only in statistical terms but also in ethical and policy implications. The responsibility lies not only with researchers and sponsors but also with journals, HTA bodies, and clinicians, who must interpret such data with calibrated skepticism.

## Conclusion: reframing real-world utility

The increasing reliance on RWD in oncology marks a pivotal shift in how we interpret clinical utility and therapeutic value. Yet, this evolution comes with inherent epistemological boundaries. Retrospective data, particularly when drawn from heterogeneous sources and uncontrolled treatment settings, must be interpreted with caution. Their strength lies not in providing definitive answers but in generating grounded hypotheses and informing prospective investigation.

The recent work by Velcheti et al.,^3^ which draws on a large cohort from the Flatiron Health database and offers meaningful observational insights into treatment patterns following progression on platinum-based chemoimmunotherapy in advanced NSCLC. However, as our analysis underscores, the unstratified nature of the data lacking adjustment for key prognostic factors like PD-L1 status or disease burden limits the granularity and actionable fidelity of its conclusions. While the apparent benefit of pembrolizumab rechallenge may reflect a real therapeutic signal, it may also reflect selective patient factors not accounted for in the analytic model.

Our position is not oppositional but corrective. RWD should not be treated as a “truth-making” tool but as a catalyst for rigorous, ethically conscious dialogue. When used to construct cost-effectiveness models or to justify coverage policies via QALY and ICER estimates, unadjusted or minimally contextualized survival data may inadvertently magnify bias and obscure true comparative utility.^18–20^ The allure of using large data volumes to generate persuasive economic narratives should not override the need for critical data appraisal.

As a constructive response, we advocate for a more disciplined approach to RWD interpretation in oncology. Transparent disclosure of selection criteria, stratification by clinical variables, preregistration of analysis plans, and publication of sensitivity analyses should be standard requirements in both academic and industry-sponsored RWD publications. Moreover, regulatory and HTA bodies must distinguish between observational insight and evidence that warrants policy action. In value-based oncology, more data is not always better; only better-framed data can drive responsible decisions.^21–23^

Future efforts should prioritize improving the granularity and clinical depth of real-world databases such as Flatiron, particularly as ‘big data’ continues to proliferate in oncology decision-making.

## Author contributions

Arif Hakan Önder (Conceptualization, Investigation, Methodology, Supervision, Validation, Writing—original draft), and Mehmet Mutlu Çatlı (Formal analysis, Investigation, Validation)

## Funding

None declared.

## Conflicts of interest

None declared.

## Data Availability

No new data were generated or analyzed for this Viewpoint. The arguments presented are based on previously published studies and publicly available literature cited in the manuscript.
